# In Vivo Wound Healing and In Vitro Anti-Inflammatory Activity Evaluation of *Phlomis russeliana* Extract Gel Formulations

**DOI:** 10.3390/molecules25112695

**Published:** 2020-06-10

**Authors:** Mehmet Evren Okur, Ayşe Esra Karadağ, Neslihan Üstündağ Okur, Yağmur Özhan, Hande Sipahi, Şule Ayla, Benay Daylan, Betül Demirci, Fatih Demirci

**Affiliations:** 1 Department of Pharmacology, Faculty of Pharmacy, University of Health Sciences, 34668 Istanbul, Turkey; 2 Department of Pharmacognosy, School of Pharmacy, Istanbul Medipol University, 34810 Istanbul, Turkey; ayseesraguler@gmail.com; 3 Department of Pharmacognosy, Graduate School of Health Sciences, Anadolu University, 26470 Eskişehir, Turkey; 4 Department of Pharmaceutical Technology, Faculty of Pharmacy, University of Health Sciences, 34668 Istanbul, Turkey; neslihanustundag@yahoo.com; 5 Department of Pharmaceutical Toxicology, Faculty of Pharmacy, Yeditepe University, 34775 Istanbul, Turkey; ozhanyagmur@hotmail.com (Y.Ö.); handesipahi@hotmail.com (H.S.); 6 Department of Histology and Embryology, School of Medicine, Istanbul Medipol University, 34810 Istanbul, Turkey; sayla@medipol.edu.tr (Ş.A.); benaydaylan94@gmail.com (B.D.); 7 Faculty of Pharmacy, Anadolu University, 26470 Eskişehir, Turkey; betuldemirci@gmail.com; 8 Department of Pharmacognosy, Faculty of Pharmacy, Eastern Mediterranean University, Famagusta, 99628 Famagusta, Cyprus

**Keywords:** *P. russeliana*, bioactivity-guided fractionation, wound healing, anti-inflammatory, phytochemical analyses, mice

## Abstract

The air-dried aerial parts of *Phlomis russeliana* (Sims) Lag. Ex Benth. was extracted by methanol and fractionated by *n*-hexane, dichloromethane, and ethyl acetate, respectively. The wound healing properties of *P. russeliana* extract gel was evaluated using the in vivo excisional wound model using Balb-c mice. Initially, the *P. russeliana* methanol extract showed LOX inhibitory activity at IC_50_ = 23.2 µg/mL, whereas the DPPH^•^ assay showed IC_50_ = 0.89 mg/mL, and the ABTS^•^ assay showed IC_50_ = 0.99 mg/mL, respectively. In addition, a remarkable anti-inflammatory activity was observed in the cell culture assay. Thereafter, activity-guided fractionation was performed by LOX enzyme inhibition assays, and the structures of the two most active fractions were revealed by both GC–FID and GC/MS analyses, simultaneously. Phytol and 1-heptadecanoic acid were characterized as the active constituents. Moreover, the *P. russeliana* extract gel formulation was applied for in vivo tests, where the new gel formulation supported the in vitro anti-inflammatory activity findings. As a conclusion, this experimental results support the wound healing evidence based on the ethnobotanical application of *Phlomis* species with further potential.

## 1. Introduction

*Phlomis* L. species of the Lamiaceae usually have aromatic characteristics, while their aerial parts have a distinctive taste. The Turkish *Phlomis* species are used in traditional medicine mainly as tonic, appetizer, carminative, and for wound healing properties also in Turkey [1,2]. Furthermore, there are immunosuppressive, anti-inflammatory, antimicrobial, and free radical scavenging activities of *Phlomis* species among others are reported [2,3].

*P. russeliana* essential oils comprise mainly sesquiterpenes and aliphatic compounds such as germacrene D, β-caryophyllene, hexadecenoic acid, and pentacosane [4]. The activity, chemistry, and ethnobotanical uses of this species were both compiled in a review [2]. The phytochemical profile of *P. russeliana* was analyzed by HPLC–PAD–MS and forsythoside B, verbascoside, samioside, isoverbascoside, leucosceptoside A, and leucosceptoside B were detected from aerial parts of this plant [5]. Moderate antimicrobial and anticancer activity of *P. russeliana* methanol extract were reported by Alpay and co-workers [6]. The bioactivity, chemistry and ethnobotanical use of this species was received extensively [2].

Wounds represent a major global health challenge, which put much economic, financial, and social stress on health institutions, health professionals, patients, and their families [7]. For centuries, plants have been used in tradition to treat and prevent diseases. Many plant preparations were used for wound repairing such as *Aloe vera* [8], *Marrubium vulgare* L. leaves [9], and *Elaeis guineensis* Jacq [10], among others. Wound healing is a natural body response when the skin is damaged by four programmed phases which are namely hemostasis, inflammation, proliferation, and remodeling [11]. Wound healing is a complex process since many factors are attributed. Wounds are easily colonized by bacteria or fungi hindering healing. Thus, clinicians recommend the use of topical antimicrobials in order to control the potential infection of deeper body tissues and blood circulation, which can cause sepsis [12]. Nonetheless, some topical antibiotics may show cytotoxicity delaying wound healing. Furthermore, antibiotic resistance may also complicate wound disinfection and healing. Hence, the use of medicinal plant preparations can be beneficial since they have biocompability, wound healing, and antimicrobial properties.

The purpose of this present study was to evaluate and determine the pharmacological activities of the herbal preparations of *P. russeliana,* which is known to be used in Anatolian folk medicine in wounds. For the identification of the active fraction(s), the air-dried aerial part of the plant material was extracted by methanol and fractionated by *n*-hexane, dichloromethane, and ethyl acetate, respectively. The phenolic constituents were characterized by the Folin–Ciocalteu method; antioxidant activity was performed by ABTS^•^ and DPPH^•^ scavenging assays, respectively. In vitro anti-inflammatory activity was evaluated both by LOX enzyme inhibition, and cell culture assays. For confirmation, the wound healing properties of *P. russeliana* extract gel formulation was studied by in vivo animal tests. To the best of our knowledge, this is the first study on the in vitro and in vivo biological activities, especially on the wound healing properties of the *P. russeliana* extract rooting to Turkish ethnobotanical uses.

## 2. Results

### 2.1. Antioxidant Activity and Total Phenolic Content

In the present study, the antioxidant activity of the *P. russeliana* methanol extract was analyzed by DPPH^•^ and ABTS^•^ free radical assays. A calibration curve was drawn with the results obtained in decreasing concentrations (0.5 mg/mL and 0.2 mg/mL) starting from 1 mg/mL, and the respective IC50 values were calculated thereof. The *P. russeliana* methanol extract showed relatively less antioxidant activity against DPPH^•^ (IC_50_ = 0.91 mg/mL) and ABTS (IC_50_ = 0.98 mg/mL) radicals compared to the standard ascorbic acid (0.002 mg/mL) and Trolox (0.015 mg/mL), respectively. Furthermore, the *P. russeliana* extract showed 3996 mg of GA/100 g extract corresponding to the total phenolic amounts, resulting in relatively moderate antioxidant activity on the in vitro ABTS^•^ and DPPH^•^ evaluations. The use of scavengers to the injured wound site was reported as effective in inflammatory conditions and wound healing [13]. The effects were attributed to the antioxidant potential, which is related to the development of matured collagen fibers and fibroblasts supporting enhanced angiogenesis [14]. Previous studies showed the antioxidant potential of *P. russeliana* extracts using the DPPH radical assay supporting our findings [6,15].

### 2.2. Antimicrobial Activity

In this present work, the antimicrobial activity of *P. russeliana* methanol extract was initially tested against human pathogenic microorganisms such as *Pseudomonas aeruginosa* (ATCC 10145), *Escherichia coli* (NRLL B-3008), *Staphylococcus aureus* (ATCC 6538), *Helicobacter pylori* (ATCC 43504), *Staphylococcus epidermidis* (ATCC 14990), and *Candida albicans* (ATCC 90028). Table 1 shows the antimicrobial activity of *P. russeliana* MeOH extract and the standards versus the tested pathogenic microorganisms. The *P. russeliana* MeOH extract showed no remarkable antimicrobial activity against the tested human pathogenic microorganisms at the tested (10 mg/mL) concentrations except *H. pylori*. However, a minimum inhibitory concentrations (MIC) value of *P. russeliana* methanol extract of 5 mg/mL against *H. pylori* was determined (Table 1). Among the analyzed bacteria, *H. pylori* was relatively more sensitive to the extract, given that the others appeared to be resistant. *Phlomis* species are rich in essential oil content. As already reported in previous studies [2,4,6,16], the apolar fractions and essential oils of the *Phlomis* species are also remarkable for their antimicrobial activity. In a study conducted by our group with *P. russeliana* essential oil, the effectiveness of this essential oil against *Bacillus cereus* and *Aeromonas hydrophila* was observed [4]. In another previous study on *P. russeliana* polar extracts, antimicrobial effects against the pathogens *S. aureus, Proteus vulgaris, Salmonella typhi, Kluyveromyces fragilis, Rhodotorula rubra, Debaryomyces hansenii, Staphylococcus pyogenes,* and *Klebsiella pneumonia* were reported [6,16].

### 2.3. LOX Enzyme Inhibition Assay

*P. russeliana* methanol extract was investigated for its biological evaluation by LOX inhibition assay. The tested extract showed moderate LOX inhibitory activity (IC_50_ = 23.2 ± 3.9 µg/mL) when compared to standard NDGA (IC_50_ = 3.2 ± 0.9 µg/mL, Table 2). Additionally, these obtained results were supported by the in vitro cell culture assays. Bioactivity-guided fractionation was performed over LOX enzyme inhibition. Sub-extracts and fractions were also evaluated for LOX enzyme inhibition. The IC_50_ value of *n*-hexane extract was calculated as 11.8 ± 1.9 µg/mL, and the IC_50_ values of dichloromethane and ethyl acetate extracts were calculated as 17.6 ± 2.1 and 23.9 ± 2.8 µg/mL, respectively. Nine different fractions (Pr-H1 to Pr-H9) from *n*-hexane extract were also evaluated for LOX enzyme inhibition and LOX enzyme inhibition was found highest in the bioactive Pr-H6 fraction (9.9 ± 0.9 µg/mL, Table 2). Seven different sub-fractions (Pr-H6.1 to Pr-H6.7) from Pr-H6 were also evaluated for LOX enzyme inhibition, and significantly significant LOX enzyme inhibition was observed in two of these seven fractions (Pr-H6.3 (4.1 ± 1.1 µg/mL) and Pr-H6.4 (4.9 ± 1.3 µg/mL)). As already well known, lipoxygenases are the key players in the formation of various physiologically active compounds of the leukotriene, lipoxin, and oxylipin families, by regulating cell metabolism and apoptosis. Moreover, these enzymes are among the most important targets for inhibition, since have been reported to play a role also in the wound healing process. Several studies recently revealed that the absence of 5-lipoxygenase leads to faster skin wound closure [17,18].

### 2.4. Bioactivity-Guided Fractionation and Chemical Characterization

After sub-extracts were prepared with *n*-hexane, dichloromethane, and ethyl acetate, respectively, fractionation was performed by open column chromatography over bioactive *n*-hexane sub-extract. Pr-H1, Pr-H2, Pr-H3, Pr-H4, Pr-H5, Pr-H6, Pr-H7, Pr-H8, and Pr-H9 were obtained after column chromatography. After all the fractions were evaluated for LOX enzyme inhibition, fractionation was continued on the bioactive fraction, Pr-H6, and two highly active subfractions (Pr-H6.3 and Pr-H6.4) were obtained from Pr-H6 (Scheme S1, in Supplementary Files).

The *P. russeliana* active fractions, which were obtained from *n*-hexane sub-extract, were analyzed by using GC-FID and GC-MS to determine the bioactive constituents, which were identified as 1-heptadecanol (10.3–20.4 %) and phytol (34.5–78.3 %). According to previous work on *P. russeliana* essential oils, mainly sesquiterpenoids such as β-caryophyllene, and germacrene D were characterized as main constituents [4]. Also, diterpenes are an important group of compounds with anti-inflammatory activities [19,20]. Phytol is an acyclic diterpene alcohol, commonly found in the composition of various oils [21,22,23], which was found among the bioactive metabolites of *P. russeliana n*-hexane sub-extract, associated with anti-inflammatory activity [24]. Thus, in the present study, the remarkable anti-inflammatory activity of the bioactive fraction may be influenced from the phytol content.

### 2.5. Wound Healing, Analgesic and Anti-Inflammatory Activity by Cell Culture Method

#### 2.5.1. Cell Culture

**Figure 1:**
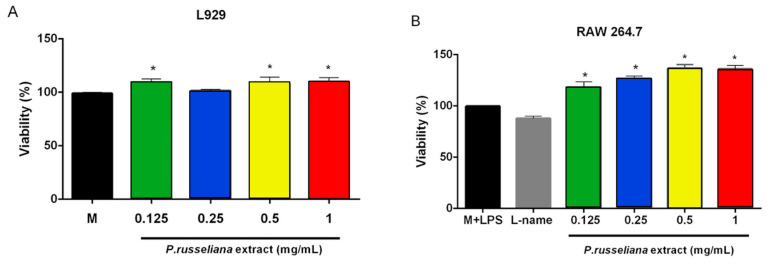
Effects of *P. russeliana* extract studied at different concentrations on L929 cell viability (**A**) and RAW264.7 cell viability (**B**). L-name: *N*-(γ)-nitro-*L*-arginine methyl ester (* *p* < 0.05).

The biocompatibility of the *P. russeliana* methanol extract was evaluated on RAW 264.7 cell lines and L929 fibroblast on cells by MTT test after treatment with various concentrations of *P. russeliana* extract for 24 h. As it is shown in Figure 1*, P. russeliana* extracts did not exhibit a cytotoxic effect on the tested cells at 0.125–1 mg/mL concentrations. In addition, a significant dose-dependent increase in RAW 264.7 cell proliferation for 0.125–0.5 mg/mL of the extract as shown in Figure 1B was observed.

#### 2.5.2. Analgesic and Anti-Inflammatory Activity

The inflammation phase on the wound healing process plays a major role in activating the inflammatory cytokines and chemokines and in recruiting macrophages which could improve the wound healing rate [25]. However, inflammation, which might occur, also damages nearby tissues. Consequently, anti-inflammation is beneficial for wound treatment.

**Figure 2:**
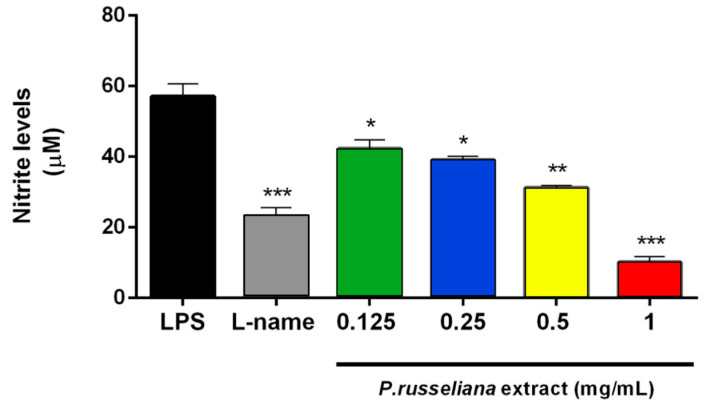
Effects of *P. russeliana* extract on nitrite production in RAW264.7 cells stimulated with 1 µg/mL of LPS. LPS: Control group treated with 1 µg/mL LPS. Statistical significances were indicated for each compound vs. LPS (* *p* < 0.05, ** *p* < 0.01, *** *p* < 0.001).

Furthermore, macrophages react in inflammatory responses through the generation of various cytokines. The activated macrophages act regarding pathogen invasion via the release of various pro-inflammatory cytokines and inflammatory mediators such as nitric oxide [26]. The *P. russeliana* extract was tested at 0.125–1 mg/mL concentrations for their inhibitory activities against lipopolysaccharide (LPS)-induced nitrite production in RAW 264.7 cells. The extract exerted anti-inflammatory activity dose-dependent fashion. The comparison of anti-inflammatory activity of the extract with the reference substance NOS inhibitor L-name (0.1 mg/mL), is shown in Figure 2.

The results revealed that the extract may work as an efficient anti-inflammatory agent by inhibiting the production of NO. The inhibition of NO production in macrophages could possibly be due to the presence of phenols, since various studies demonstrated that phenols can act as anti-inflammatory agents, and they play a key role in oxidative stress and inflammation [26].

Pain is a highly unpleasant physical sensational and emotional experience associated with actual or potential tissue damage. The unresolved wound pain can have a negative impact on wound healing by delaying the process [27].

**Figure 3:**
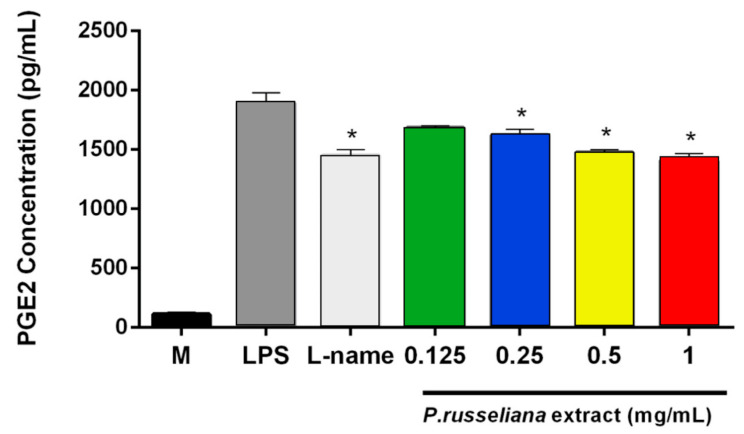
Analgesic effect of *P. russeliana* extract on LPS-induced production of PGE_2_. LPS: Control group treated with 1 µg/mL LPS. L-name: *N*-(γ)-nitro-*L*-arginine methyl ester. Statistical significances were indicated for each compound vs. LPS (* *p* < 0.05).

In this present work, the possible analgesic potency of the *P. russeliana* extract is remerkable since it can serve both as a wound healing and analgesic agent. It is well established that the release of high levels of prostaglandins (PGE_2_) is produced in response to injury or infection, and may causes inflammation, which is associated with the symptoms of redness, swelling, pain, and fever [28]. Thus, herein, the analgesic activity on PGE_2_ productions was evaluated for the extract doses with anti-inflammatory activity. As in Figure 3, both *P. russeliana* extract (0.25–1 mg/mL) and N(gamma)-nitro-L-arginine methyl ester (L-NAME; 0.1 mg/mL) inhibited LPS-induced production of PGE_2_. Therefore, reduction/termination of the persistent inflammation and elimination of free radicals by the introduction of an antioxidant could be an important strategy to improve healing [29].

### 2.6. In Vivo Wound Healing

As it is well known, the wound healing process comprises three different phases. The first phase is when the inflammation occurs; consequently, the neutrophils and macrophages accumulate to the lesion location. The following stage is the proliferation, when re-epithelization, angiogenesis, fibroplasia, and granulation tissue formation happen. Finally, in the remodeling phase, the blood vessels amount (neovascularization) is reduced and the neocollagenesis course occurs. Subsequently, the wound is diminished. The target of wound healing is either to reduce the healing time or to avail undesired complications such as scarring [30].

**Figure 4:**
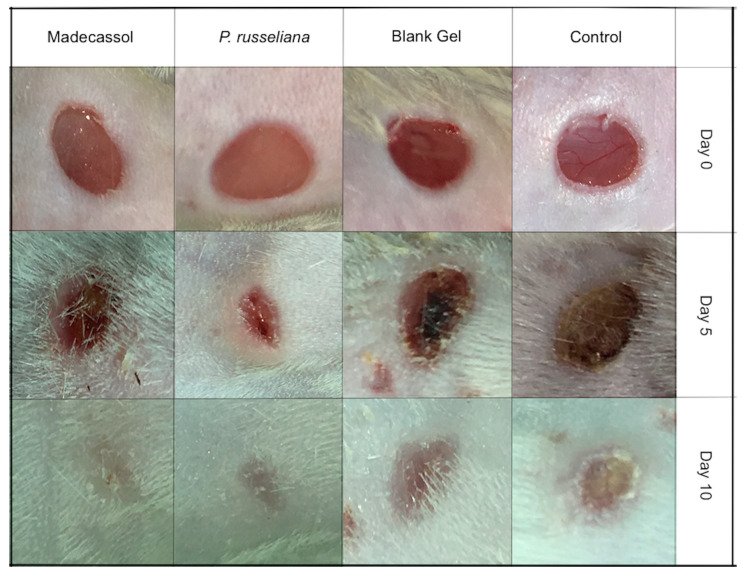
Macroscopic visualization of test samples: control, blank gel, Madecassol, *P. russeliana* extract test groups at days 0., 5. and 10.

**Figure 5:**
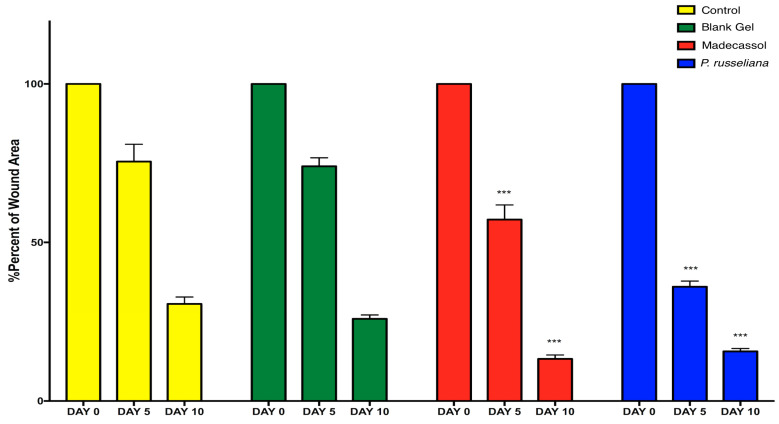
Healing percentages of the wound area in each test group (control, blank gel, Madecassol, *P. russeliana* extract gel). Statistically significant, compared to control groups; *** *p* < 0.001.

Figure 4 and Figure 5 depict the macroscopic healing rates of wounds on the studied animals, the wound surface size of the studied group was monitored on days 0, 5, and 10, respectively. The control, blank gel and the control Madecassol^®^ showed wound scabs on day 5. on day 10, *P. russeliana* extract gel and Madecassol^®^ depicted healing since a characteristic lesion can be observed.

In Figure 5, the percentage of wound healing rates can be observed comparatively. More specifically, on Day 5, *P. russeliana* gel and Madecassol^®^ showed similar wound area percentages, which were relatively more, compared to control and blank gel. In addition, on Day 10 significant reductions in the wound surface were observed for for the *P. russeliana* gel and Madecassol^®^ since both wounds were almost recovered.

### 2.7. Histological Evaluation of Wound Healing

**Figure 6:**
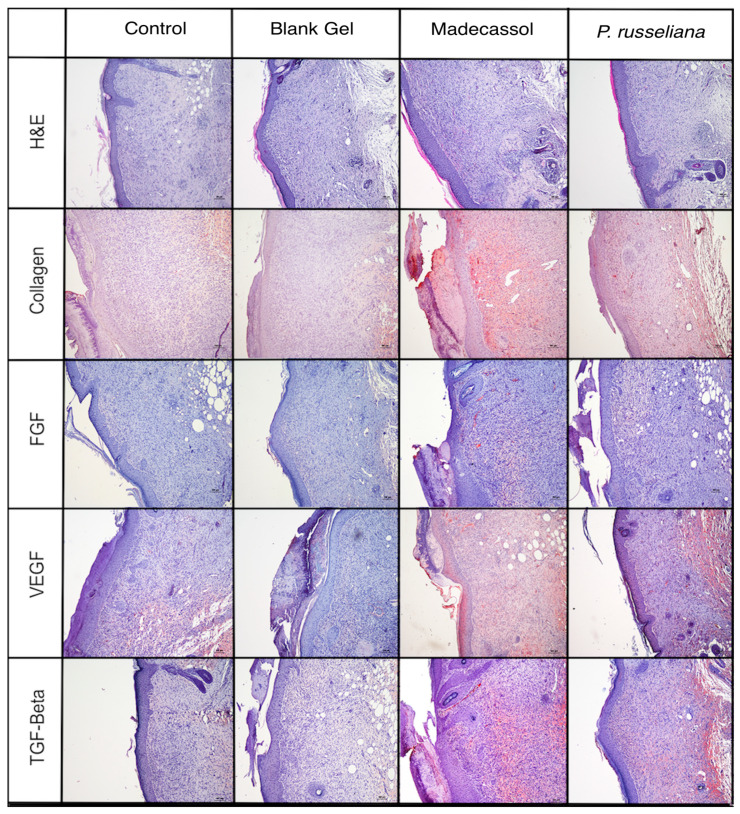
Histopathological view of injured tissues of the untreated (control), blank gel, extract gel, and Madecassol groups on the 10th day after wound incision (magnification 10×).

The proliferative phase presents various stages, such as angiogenesis, epithelialization, the formation of granulation tissue, fluid loss protection as well as bacterial invasion. In general, angiogenesis impacts several processes including wound healing, embryonic development, tumor growth, and chronic inflammation. Herein, angiogenesis, granulation tissue thickness, and epidermal–dermal regeneration, TGF-b, vascular endothelial growth factor (VEGF), FGF, and collagen were examined separately. The histopathological observation of the wound area by hematoxylin and eosin (H&E) and immune-histochemical staining is shown in Figure 6.

**Figure 7:**
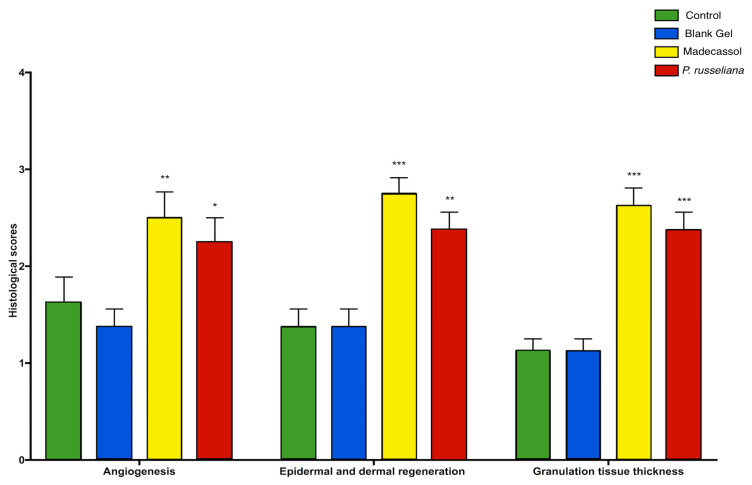
Histological granulation scores; tissue thickness, angiogenesis, and epidermal–dermal regeneration of control, blank gel, Madecassol, *P. russeliana* extract gel groups. Statistically significant compared to control groups; * *p* < 0.05, ** *p* < 0.01, *** *p* < 0.001.

Figure 7 showed both that Madecassol and *P. russeliana* extract gel significantly (*p* < 0.001) affect angiogenesis, epidermal, and dermal granulation as well as granulation tissue thickness compared to control and blank gel. In addition, the vast formation of blood vessels was depicted again both for Madecassol and *P. russeliana* extract gel (*p* < 0.001) in comparison results suggest that the group treated with *P. russeliana* extract gel demonstrate new blood vessel formation similarly to Madecassol treated group.

Besides angiogenesis, the improvement of dermal and epidermal regeneration, which consists of well-structured epithelial layers with an absence of crusting or intraepithelial inflammatory cells, is essential for wound healing. Herein, great dermal and epidermal regeneration was revealed after the treatment of the groups with Madecassol^®^ and *P. russeliana* gels compared to control and blank gel (Figure 7). It can be argued that the *P. russeliana* gel is similarly effective as Madecassol^®^ gel on epithelialization, granulation tissue thickness, and angiogenesis significant for the evaluation of wound healing. Moreover, the observed in vivo wound healing can be associated with the presence of phytol, which can accelerate and enhance the wound healing [30].

**Figure 8:**
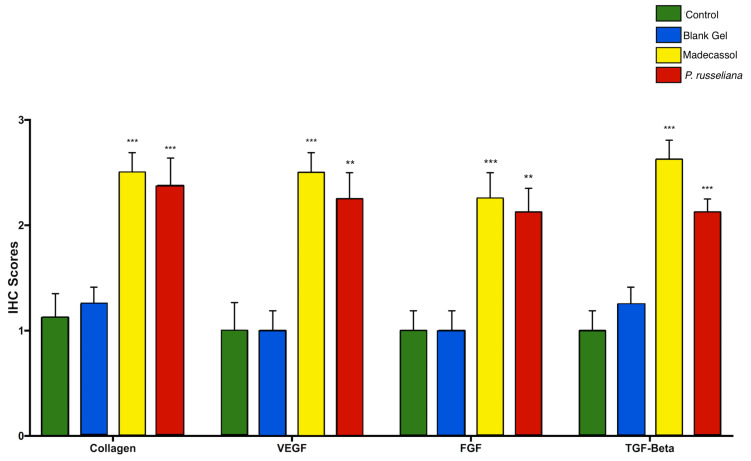
Comparison of immunohistochemistry; collagen, TGF-Beta, VEGf, FGF wound healing scores among the test groups. Statistically significant compared to control groups; ** *p* < 0.01, *** *p* < 0.001.

Figure 8 depicts the comparison of collagen formation scores per group. Collagen is structurally and functionally a key protein of the extracellular matrix which is also involved in scar formation during the healing of connective tissues. Collagen deposition and can supply the strength and integrity that wounds need and can provoke re-epithelization. The strength of the recovered wound tissue is a result of the remodeling of collagen and the formation of stable intra and intermolecular cross-linking to form fiber [31]. Herein, both Madecassol (*p* < 0.001) and *P. russeliana* gel (*p* < 0.001) treated groups demonstrated greater statistically collagen formation (over two-fold collagen formation) compared to blank and control groups.

Figure 8 also exhibited the comparison of immunohistochemistry scores for TGF-b. The TGF-b, platelet-derived growth factor (PDGF), fibroblast growth factor (FGF), epidermal growth factor (EGF) as well as vascular endothelial growth factor (VEGF) are found in reduced levels on chronic wounds. In further, chronic wounds show decreased levels of interleukins 1 and 6 (IL-1 and -6) and tumor necrosis factor-a (TNF-a) [32]. In addition, TGF-b has shown potent characteristics as wound healing growth factor and can enhance wound healing rate and avoidance of scarring. In this study, it was shown (Figure 8 that the levels of TGF-b in Madecassol (*p* < 0.001) and *P. russeliana* gel groups (*p* < 0.001) were significantly greater than control and blank gel groups.

Moreover, Figure 8 demonstrated the VEGF scores of the studied groups. VEGF is quite significant since it is involved in granulation tissue formation and thus further enhanced angiogenesis and wound healing [33]. It revealed that Madecassol (*p* < 0.001) and *P. russeliana* gels (*p* < 0.01) had a significant increase of VEGF scores (more than two-fold increase) in comparison with the control group and blank gel groups as observed in Figure 8.

The comparison of immunohistochemistry scores for FGF, resulted in the multi-potentiality of basic FGF, which plays an important role at the site of injury and is known to promote wound healing, as also confirmed in Figure 8. FGF was reported to enhance the proliferation of human periodontal ligament cells in the oral area [34]. In this study, it was shown that the expression of FGF in Madecassol (*p* < 0.01) and *P. russeliana* gel groups (*p* < 0.001) was significantly greater than control and blank gel groups.

## 3. Materials and Methods

### 3.1. Materials

Carbopol and Glycerine were purchased from Sigma, Germany. Lipoxygenase (1.13.11.12, type I-B, Soybean), linoleic acid, and LPS (lipopolysaccharide from *Escherichia coli* 0111:B4) were purchased from Sigma (St. Louis, MO, USA). The RAW 264.7 murine macrophage cell line and L929 healthy mouse fibroblast were purchased from ATCC (Gaithersburg, MD, USA) and DMEM was purchased from Gibco (New York, NY, USA). All the other chemicals and solvents were of analytical or HPLC grade.

### 3.2. Plant Material, Extraction, and Bioactivity-Guided Fractionation

*P. russeliana* was collected in June 2015 from Abant, Bolu, Turkey. Voucher specimens were deposited in the Herbarium of the Faculty of Pharmacy, Istanbul University (ISTE 115022). The air-dried aerial parts were crushed and extracted with methanol by maceration followed by filtration and evaporation (Heidolph, Darmstadt, Germany). The sub-fractions were prepared via liquid-liquid extraction using *n*-hexane, dichloromethane, and ethyl acetate, respectively.

The *n*-hexane extract (4 g) was purified using a Silica column (400 g) eluting with *n*-hexane and stepwise gradient of ethyl acetate in *n*-hexane to yield nine fractions, from Pr-H1 to Pr-H9. The Pr-H6 (250 mg) was applied to a Silica (20 g) column and eluted with *n*-hexane and a gradient of ethyl acetate in *n*-hexane to obtain further six fractions, from Pr-H6.1 to Pr-H6.6. The phytochemistry of Pr-H6.3 and Pr-H6.4 were determined by GC–MS/GC–FID.

### 3.3. GC-FID and GC/MS Analyses 

The bioactive fractions of *P. russeliana* were analyzed by an Agilent 5975 GC-MSD system. The used column was the Innowax FSC (60m × 0.25mm, 0.25 µm film thickness) using helium as carrier gas (0.8 mL/min). The GC oven temperature set at 60 °C for 10 min and programmed to 220 °C at a rate of 4 °C/min, and kept constant at 220 °C for 10 min and then programmed to 240 °C at a rate of 1 °C/min. The split ratio was adjusted at 40:1. The injector temperature was set at 250 °C. The mass spectra were recorded at 70 eV and the mass range was from *m/z* 35 to 450.

The GC analysis was carried out using an Agilent 6890N GC system. The temperature of FID detector was 300 °C. A simultaneous auto-injection was done on a duplicate of the same column applying the same operational conditions in order to obtain the same elution order with GC/MS. The relative percentage amounts (%) of the separated compounds were calculated from FID chromatograms.

The identifications of the oil components were performed by comparison to their relative retention times (RRT) with those of authentic samples or compared to their relative retention index (RRI) to series of *n*-alkanes. The computer matching of the separated compounds against commercial (Wiley GC/MS Library, MassFinder Software 4.0) and the in-house “Başer Library of Essential Oil Constituents” built up by genuine compounds and components from literature previous reported [3,4,21]. 

### 3.4. In Vitro Biological Assays

#### 3.4.1. Antioxidant Activity and Total Phenolic Content

##### Radical Scavenging Activity by DPPH Method

Radical scavenging activity of the extract was detected by its capacity of bleaching the stable radical DPPH [35]. The reaction mixture comprised of 100 µM DPPH^•^ in methanol and *P. russeliana* MeOH extract. The mixture’s absorbance was determined at 517 nm by a UV spectrophotometer (UV-1800, Shimadzu, Japan) at room temperature. The radical scavenging activity percentage inhibition (%) was obtained as follows:

% = [(Absorbance _control*_ – Absorbance _test sample_) / Absorbance _control*_)] × 100 (1)

(*Control; Ascorbic acid)

##### ABTS Radical Scavenging Assay

The antioxidant capacity of the *P. russeliana* MeOH extract was determined according to previous literature [36]. The solution was prepared by mixing potassium persulfate and ABTS^•^ and allowing them to react at room temperature for 16 h in the dark. The absorbance was set at 734 nm. The study was performed in triplicate. The percentage inhibition (%) was calculated as:

% = [(Absorbance _control*_ – Absorbance _test sample_) / Absorbance _control*_)] × 100 (2)

(*Control; Trolox)

##### Total Phenolic Content

Folin–Ciocalteu method was used for the determination of the total phenolics content of *P. russeliana* MeOH extract. Folin-Ciocalteau’s reagent and Na_2_CO_3_ were mixed with extract and allowed to incubate at 45 °C for 15 min. The absorbance was determined at 765 nm at room temperature. The total phenolic ingredient was measured from a linear calibration curve (R^2^ = 0.9936) [37].

#### 3.4.2. Antimicrobial Activity

The in vitro antimicrobial activity was evaluated by determining the minimum inhibitory concentrations (MIC) via the broth microdilution protocol according to the Clinical and Laboratory Standards Institute [38]. The commonly found microorganisms on colonized wounds were studied; *S. aureus, P. aeruginosa*, *S. epidermidis,* and *E. coli* strains were grown in Mueller Hinton Broth (MHB, Merck, Germany); *C. albicans* species were grown in RPMI broth at 37 °C in aerobic conditions for 24 h. Bacteria suspension was adjusted to 0.5 McFarland turbidity standards (corresponding to 10^8^ colony-forming units (CFU)/mL) with sterile saline.

The *H. pylori* strain was grown in Brucella broth (Sigma Aldrich) at 37 °C under anaerobic atmosphere for 24 h. After incubation, 100 µL of 1:10 diluted and transferred to the microplates with an inoculum of 2 × 10^7^ CFU/mL of *H. pylori* [39]. The diluted bacterial suspensions were added in each well and then incubated at 37 °C for 24 h.

All samples were prepared in dimethyl sulfoxide (DMSO). MIC (as μg/mL) was defined as the lowest concentration that inhibited visible growth. The MIC was screened and the results were compared to positive controls as shown in Table 1. Each experiment was replicated thrice.

#### 3.4.3. LOX Enzyme Inhibition Assay

The 5-lipoxygenase assay (5-LOX inhibiting activity) was measured by modifying the spectrophotometric method [40]. Potassium phosphate buffer (1,94 mL;100 mM; pH 9.0), 40 µL of test solution, and 20 µL of lipoxygenase solution were mixed and incubated for 10 min at 25 °C. The reaction was initiated by the addition of 10 µL linoleic acid solution, the change of absorbance at 234 nm was followed for 10 min.

The test sample and the Nordihydroguaiaretic acid (NDGA, positive control) were dissolved in MeOH. All the kinetic experiments were performed in quartz cuvette as triplicate. The concentration of the extracts was calculated for IC_50_ value. All test and control assays were corrected by blanks for non-enzymatic hydrolysis. The absorbance change per minute was determined. The percentage of inhibition was calculated as the absorbance change per minute of enzyme activity (without any inhibitor) compared to absorbance change per minute of the test sample.

### 3.5. In Vitro Anti-Inflammatory, Analgesic, and Wound Healing Evaluation of P. russeliana MeOH Extract

#### 3.5.1. Cytotoxicity Assay

The RAW 264.7 and L929 healthy mouse fibroblasts were cultured in DMEM supplemented with 10% FBS and 1% penicillin (10.000 units/mL) and streptomycin (10.000 µg/mL) at 37 °C at a humidified atmosphere of 5% CO_2_. For the analysis of the cytotoxicity, MTT colorimetric assay was used [41]. The plated RAW 264.7 cells and L929 cells were treated with various concentrations of *P. russeliana* extract (0.125, 0.250, 0.5, and 1 mg/mL). After incubation for 24 h, the medium was removed and MTT assay reagent (0.5 mg/mL) was added to wells and cells were incubated at 37 °C. After 2 h incubation, the cell culture medium was removed and isopropanol (100 μL) was added to dissolve the formazan crystals. The plates were then read by a microplate reader (Thermo Multiscan Spectrum, Vantaa, Finland) at a wavelength of 570 nm spectrophotometrically. The percentage of cell viability was calculated according to the formula:

Viability % = [(Absorbance _treatment group_) (Absorbance _control_)] × 100 (3)

#### 3.5.2. Anti-Inflammatory Activity

The anti-inflammatory activity of *P. russeliana* extract was assessed by determining the stable end product of nitric oxide (NO), Nitrite levels in the culture medium using the Griess reagent (0.1% *N*-(1-naphthyl) ethylenediamine dihydrochloride in 5% phosphoric acid and 1% sulfanilamide) [42]. The RAW 264.7 cells transferred in a 96-well plate at the density of 5 × 10^4^ cells/well and incubated for 24 h at 37 °C in 5% CO_2_. The cells were pre-treated with the concentrations of *P. russeliana* extract (0.125, 0.25, 0.5, and 1 mg/mL) for 2 h. Afterwards, the cells were stimulated with 1 μg/mL of LPS for 22 h. Then, the cell culture supernatant was collected. Fifty microliters of the supernatant were mixed with the equal volume of Griess reagent and the solution was incubated at room temperature for 10 min in the dark. The absorbance was measured at 540 nm with a microplate reader (Multiscan Ascent, Vantaa, Finland). The nitrite levels in samples were established using a sodium nitrite standard curve. As positive control, the N(gamma)-nitro-L-arginine methyl ester (L-Name; 0.1 mg/mL) was used.

#### 3.5.3. Analgesic Activity

The analgesic activity was analyzed for the samples with greater anti-inflammatory activity. The detection of Prostaglandin E_2_ (PGE_2_) levels in collected RAW 264.7 cell culture supernatants were achieved using the commercially available quantitative enzyme-linked immunosorbent assay (ELISA) kit (Abcam PGE_2_ ELISA Kit, Cambridge, UK).

### 3.6. Preparation of Topical Extract Gel

The extract loaded gel was formulated by mixing Carbopol aqueous gel and hydroxypropyl cellulose gel. Firstly, 0.5 g Carbopol (dispersed in water), 7 g glycerin, and 20 g isopropyl alcohol were blended with slowly stirring using a mechanical stirrer, at 25 °C and 3.5 g of triethanolamine in water was transferred. The mixture was further filled with water up to 100 g weight. Following, the blend was stirred constantly until the formation of a clear gel. In addition, a hydroxypropyl cellulose gel (2%) was prepared. The Carbopol gel and hydroxypropyl cellulose gel were mixed (50:50) ratio and 5% of the extract was added following by gently stirring. Similarly, a blank gel was also prepared.

### 3.7. In Vivo Experiments

#### 3.7.1. Animals

The animal experiment was performed according to the local ethics committee regulations (Istanbul Medipol University Institutional Animals Ethical Committee, Approval No: 2018-09). The European Community guidelines as accepted principles for the use of experimental animals, were adhered to.

Balb-c mice (25–28 g) were procured from Istanbul Medipol University, MEDITAM, Istanbul, Turkey. The mice were kept in regular cages with food and water ad libitum, at room temperature (20 ± 2 °C) with artificial light from 7.00 am to 7.00 pm.

#### 3.7.2. Wound Model and Experimental Groups

Four groups of seven animals (*n* = 7) from the totally used twenty-eight mice were assigned randomly;

1. Untreated (Control) group;
2. Blank gel (Vehicle) group;
3. *P. russeliana* extract gel group;
4. Madecassol (Bayer, Switzerland, Standard drug) group.

Animals were anesthetized via mixture of ketamine (80–100mg/kg, intraperitoneal (i.p.)) and xylazine (10 mg/kg, i.p.). Afterwards, the back of the mice was shaved with an electric razor and a povidone-iodine solution was applied to dorsal skin. Full-thickness excisional skin wounds were created bilaterally in the dorsal skin with a 5-mm round skin biopsy punch. The gels were topically applied to the wound’s surface every day for 10 days.

#### 3.7.3. Macroscopic Wound Healing

The day when wounds were made was designated as Day 0, and the process of wound healing was observed from then until Day 10 after wounding. Wound photographs were taken using a digital photo machine (Canon Inc., Tokyo, Japan) with an internal scale of each wound at days 0, 5, and 10 to measure wound contraction. The pictures of the wounds were documented at a 90° angle to the plane of the wound. Wound surface areas were calculated using an image analyzer software (Image J.2.0 software, Bethesda, MD, USA) to assess wound healing ratios of per group.

#### 3.7.4. Histology and Immunohistochemistry

Mice were sacrificed on Day 10 following the wounding and the whole wound with a margin of around 5 mm of ambient unwounded skin was excised for histological evaluation. All samples were fixed in 10% neutral formalin. After 24 h, the biopsies were bisected, embedded in paraffin, and sectioned in 5 µm thick layers

The 5 µm thick sections were mounted on glass slides, dewaxed, rehydrated with distilled water and stained with vascular endothelial growth factor (VEGF, Santa Cruz sc-7269), fibroblast growth factor FGF (Santa Cruz sc-55520), transforming growth factor beta1 (TGF-b1, Santa Cruz sc-65378), Collagen type1 alpha1 (COL1A1, Santa Cruz sc-293182), and hematoxylin-eosin (HE) in order to be evaluated via light microscopy.

The wound was assessed according to the scoring system as previously reported [43]. Epidermal and dermal regeneration score system was: (1) Epidermal formation (poor) ≥20%; (2) Epidermal formation (incomplete) ≥40%; (3) Epithelial proliferation (moderate) ≥60%; (4) Epidermal re-modeling (complete) ≥80%. The thickness of the granulation tissue score system was; (1) thin layer; (2) moderate layer; (3) thick layer; (4) very thick layer.

The angiogenesis was evaluated by counting and identifying only mature vessels and the presence of erythrocytes in the lumen. The presence/absence of edema, thrombosis, hemorrhage, congestion, and intra/intervascular fibrin formation was evaluated to distinguish well-formed and poorly formed capillary vessels. Angiogenesis score system was: (1) High level of hemorrhage, edema, occasional congestion, and thrombosis; (2) Capillary vessels (newly formed, 3–4/site), occasional congestion, moderate edema and hemorrhage, intravascular fibrin deposition and absence of thrombosis; (3) Capillary vessels (newly formed, 5–6/site); (4) Capillary vessels (newly formed and normal-appearing >7/site).

The immune-reactivity of immune-histochemical stain was assessed via the semi-quantitative method. Five randomly selected areas were chosen for the evaluation, where the scoring system was: (0) No painting, (1) Little, (2) Medium, (3) Heavy painting [43].

#### 3.7.5. Statistical Analysis

In vitro and In vivo data were expressed as mean ± standard error of the mean (Mean ± SEM). The statistical significance between groups was analyzed by One-way ANOVA (followed by Dunnett’s post hoc test, in vivo) and Paired Samples T-Test (in vitro). The statistical analysis was carried out using GraphPad Prism 7.0 (GraphPad Software Inc., San Diego, CA, USA) and SPSS Statistics 22.0 (IBM, Chicago, IL, USA). *p* < 0.05 was suggested as statistically significant.

## 4. Conclusions

The aerial parts of *P. russeliana* were extracted with various solvents and formulated by a gel preparation, which was studied for its wound healing associated biological activities both in vitro and in vivo, to the best of our knowledge for the first time. The GC–MS and GC–FID analyses revealed the abundance of the bioactive compounds, phytol, and 1-heptadecanol, which are also associated with the antioxidant and anti-inflammatory properties. The *P. russeliana* methanol extract showed moderate anti-*Helicobacter pylori* activity, LOX inhibitory and antioxidant activity. Following in vitro tests with anti-inflammatory and analgesic effects, expected supportive results were obtained in in vivo wound healing experiments. The anti-inflammatory and analgesic activity of *P. russeliana* extract was statistically significant, supported by both in vitro and in vivo wound healing activity, suggesting that *Phlomis* species can be correlated to traditional uses. The experimental design supports the wound healing properties by scientific evidence and the further potential of the ethnobotanical application of *Phlomis* species.

## Figures and Tables

**Table 1:** Antimicrobial activity of *P. russeliana* methanol extract (MICs in mg/mL).

Samples	*E. coli*	*S. aureus*	*S. epidermidis*	*P. aeruginosa*	*H. pylori*	*C. albicans*
MeOH extract	>10	>10	>10	10	5	10
Amoxicillin	0.5	≤0.125	4	>16	≤0.125	nt
Clarithromycin	0.5	0.25	≤0.125	>16	0.025	nt
Tetracycline	16	0.25	>16	>16	0.025	nt
Ketoconazole	nt	nt	nt	nt	nt	0.25

nt: not tested.

**Table 2:** LOX enzyme inhibition of *P. russeliana* extracts and fractions (in g/mL).

Samples	IC_50_
MeOH extract	23.2 ± 3.9
*n*-hexane sub-extract	11.8 ± 1.9
dichloromethane sub-extract	17.6 ± 2.1
Ethyl acetate sub-extract	23.9 ± 2.8
Pr-H1	43.9 ± 3.9
Pr-H2	29.7 ± 2.4
Pr-H3	19.3 ± 1.7
Pr-H4	23.4 ± 3.1
Pr-H5	14.1 ± 2.3
Pr-H6	9.9 ± 0.9
Pr-H7	11.7 ± 1.8
Pr-H8	13.9 ± 3.4
Pr-H9	16.5 ± 1.5
Pr-H6.1	18.9 ± 2.8
Pr-H6.2	17.4 ± 1.6
Pr-H6.3	4.1 ± 1.1
Pr-H6.4	4.9 ± 1.3
Pr-H6.5	13.4 ± 3.1
Pr-H6.6	39.1 ± 2.3
NDGA	3.2 ± 0.9

## Supplementary Materials

The following are available online: Scheme S1: Bioactivity-Guided Fractionation and Chemical Characterization.
