# Supplementary material for: In Vivo Wound Healing and In Vitro Anti-Inflammatory Activity Evaluation of Phlomis russeliana Extract Gel Formulations
Source: Molecules. 2020 Jun 10;25(11):2695. doi: 10.3390/molecules25112695 (PMC7321211; doi:10.3390/molecules25112695)

Supplementary data:

In Vivo Wound Healing and *In Vitro* Anti-inflammatory Activity Evaluation of *Phlomis russeliana* Extract Gel Formulations,

Mehmet Evren Okur [1,*], Ayşe Esra Karadağ [2,3], Neslihan Üstündağ Okur [4], Yağmur Özhan [5], Hande Sipahi [5], Şule Ayla [6], Benay Daylan [6], Betül Demirci [7] and Fatih Demirci [7,8,*]

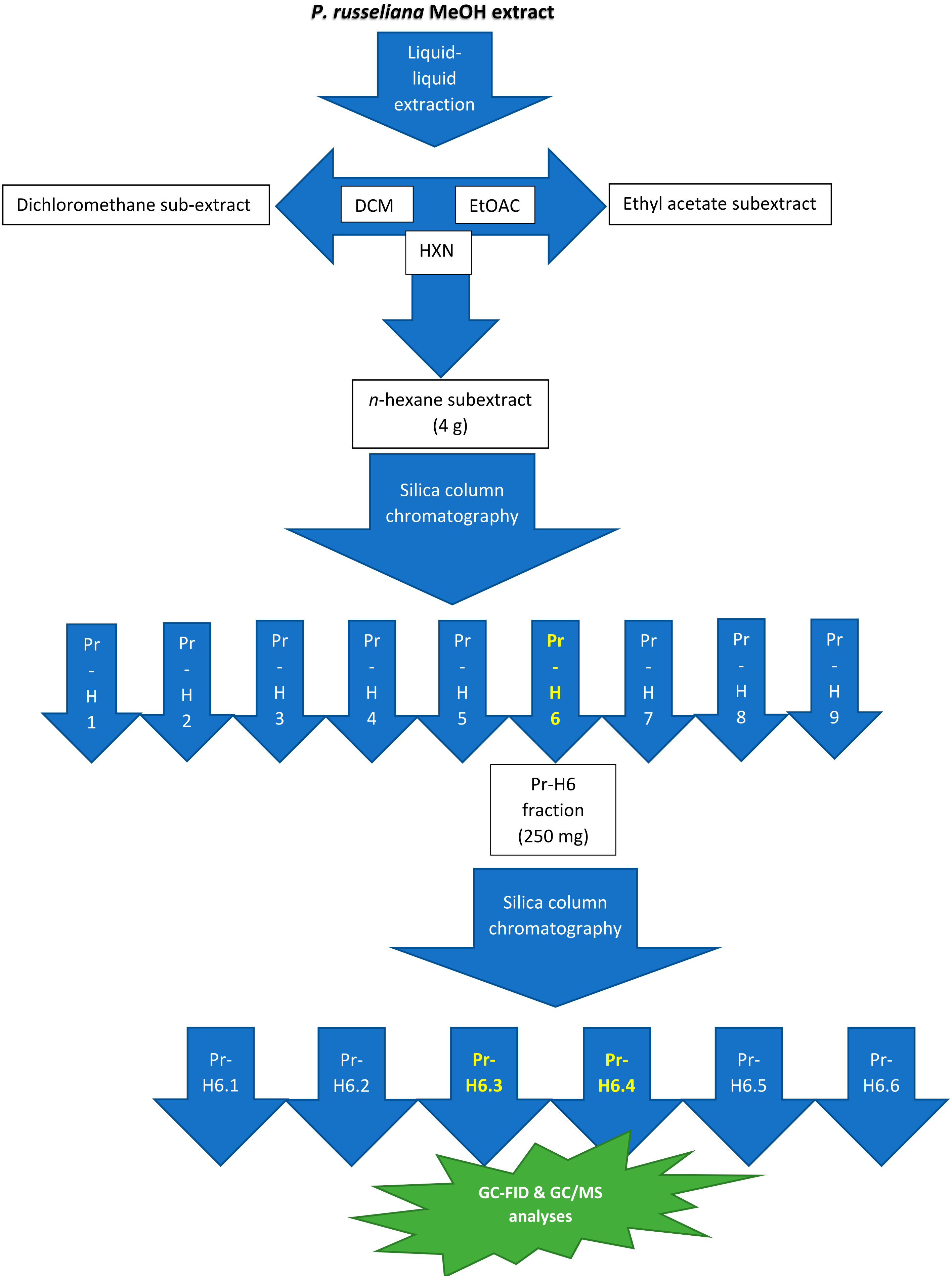

Supplement: Supplementary file 1 [file molecules-25-02695-s001.pdf]
